# Factors affecting the collection of clinical data for quality improvement at a tertiary centre in Papua New Guinea: a qualitative study

**DOI:** 10.7189/jogh.16.04125

**Published:** 2026-05-08

**Authors:** Samuel JA Robinson, Francesca Failing, Waku Albert, Nane Buni, Duta Yema, Teba Anterea, Debra Nestel, Elizabeth McLeod, Maurizio Pacilli, Ramesh Mark Nataraja

**Affiliations:** 1 Department of Paediatrics, Monash University, Melbourne, Victoria, Australia; 2 Department of Paediatric Surgery and Monash Children’s Simulation, Monash Children’s Hospital, Victoria, Melbourne, Australia; 3 Department of Surgery, Monash University, Melbourne, Victoria, Australia; 4 Department of Paediatrics, ANGAU Memorial Provincial Hospital, Lae, Papua New Guinea; 5 School of Clinical Sciences, Monash University, Melbourne, Victoria, Australia; 6 Department of Surgery, University of Melbourne, Melbourne, Victoria, Australia; 7 Department of Paediatric and Neonatal Surgery, Royal Children’s Hospital, Melbourne, Victoria, Australia

## Abstract

**Background:**

The collection of clinical audit data are important for audit, research and other quality improvement processes. Low- and middle-income countries (LMICs) face a range of challenges to effective data collection and use. In this study, we explored factors affecting the collection of clinical data at a tertiary centre in Papua New Guinea.

**Methods:**

This qualitative study was based on semi-structured individual interviews with healthcare workers at a tertiary hospital in Papua New Guinea. Questions focused on participants’ experiences and perspectives regarding the collection of clinical data. We transcribed the interviews and analysed them using reflexive thematic analysis. We collected demographics using a short survey. Reporting of the research was guided by COREQ.

**Results:**

We conducted 20 interviews with predominantly nursing staff (n/N = 15/20; 75%). Of the participants, 16/20 (80%) had previously collected data for an audit or research project. Five themes and eight subthemes were conceptualised. Themes related to organisational culture, staff workload, data documentation practices, research infrastructure and study procedures. Although participants were motivated to complete data collection, they faced significant challenges that hindered their ability to do so. While staff often had to choose between collecting data and providing patient care, data collection initiatives could also improve patient care by improving communication between healthcare workers.

**Conclusions:**

In this study, we revealed numerous factors which affect the success of clinical data collection for audit and research purposes. These factors may be considered in the design of future initiatives. This has significant implications given the public health importance of strengthening data collection in Papua New Guinea, the Pacific Islands and LMICs in other regions.

Vast amounts of data are generated in healthcare systems, with this volume growing exponentially in high-income countries over recent years [1]. When collected and used effectively, clinical data form the foundation of evidence-based medicine and are essential for benefiting patients, institutions and society. Clinical data collection is often a key component of clinical audits, in which data are compared against evidence-based standards [2,3]. Through this process, audits can help develop strategies to improve care within clinical governance frameworks. Clinical data collection also forms the basis of clinical research, which furthers evidence-based practice globally [4]. More broadly, it is essential for other quality improvement efforts, such as those relating to evaluation, public policy and advocacy.

In many contexts, clinical data collection has become integrated into routine practice [5,6]. This has been aided by the introduction of electronic health records and medical device data, which have significantly increased the amount of available data [7]. There has been an associated interest in the concept of ‘real-world data,’ a term that describes routinely collected data from a variety of sources outside controlled settings [8]. Real-world data are used to generate real-world evidence through a variety of observational studies [9].

Despite expanding opportunities for data analysis and utilisation, many settings still experience difficulties in collecting and using clinical data [10,11]. Low- and middle-income countries (LMICs) may lack the infrastructure and systems to generate and utilise real-world data [12]. Additionally, many face systemic barriers to effective data collection [13,14]. When reviewing the implementation of mortality audits in LMICs, Gondwe et al. described 23 barriers, most commonly relating to planning, training, clinical workloads and time [13]. Another review of health data use in LMICs was conducted by the United States Agency for International Development (USAID) [14]. They described nine barriers, including factors related to individuals, institutions, and the data itself. These reviews provided potential solutions, including capacity-building, system changes, and advocacy. While these insights are valuable, further investigation is necessary to optimise data collection processes in specific regions and countries.

Papua New Guinea (PNG) can benefit from a strengthened healthcare system, including improved clinical data-collection capacity for audit and research. A report on health research capacity, endorsed by the World Health Organization (WHO), ranked PNG 128 out of 180 countries [15]. As such, in this study, we aimed to explore factors affecting the collection of clinical data for audit and research purposes at a tertiary centre in PNG. The study was conducted at the Australian New Guinea Administrative Unit (ANGAU) Memorial Provincial Hospital (ANGAU hospital). ANGAU hospital is the second-largest hospital in the country, serving many of the 220 000 people in the Lae district and over one million people in the wider Morobe province [16].

To our knowledge, this is the first study of its kind in this context. Understanding factors that affect clinical data collection will subsequently guide the identification of strategies to improve these processes. In addition to benefiting data use at ANGAU hospital, these findings are likely to be relevant to similar low-resource settings.

## METHODS

### Study design

We used reflexive thematic analysis of transcribed semi-structured interviews. We conducted the study from an interpretivist perspective, seeking to understand participants’ diverse experiences of the same phenomenon [17]. More broadly, this paper contributes to a series of postgraduate studies with a realist orientation. In this orientation, qualitative research can provide insight into the ‘actual’ level of realist ontology [18]. For the reporting, we followed COREQ guidelines (Appendix S1 in the **Online Supplementary Document**) [19].

Healthcare workers at ANGAU hospital were recruited during September 2024. All healthcare workers in the department of paediatrics were eligible to participate, including nurses, health extension officers, community health workers and doctors. The study was approved by the ANGAU hospital and Monash University research ethics committees (Monash University project identification 41889).

We developed a semi-structured interview guide based on existing literature in consultation with experienced qualitative researchers (DN and RN). Questions focused on participants’ experiences with data collection for audit and research, and factors impacting data collection of this nature. This form of data collection should be delineated from the everyday documentation of clinical care and processes. There is evidently some overlap among these concepts, as documentation of clinical care is often incorporated into audit and research processes [8]. We used a short survey to collect demographic information, including gender, occupation, and experience with clinical data collection for audit and research.

We recruited potential participants through two methods – direct contact by local clinical leaders or via snowball sampling. The clinical leaders formed the ANGAU hospital portion of the collaboration and included several nurse unit managers and the acting head of department. Snowball sampling was incorporated as a flexible recruitment method, given that staff availability was highly dependent on clinical workload. Staff would usually attend interviews during shift breaks, which were determined based on patient flow. Consequently, staff were best positioned to decide on an appropriate time to attend interviews and would then inform their colleagues upon their own completion. The formal introduction to the study and informed consent process was always conducted by SR prior to commencing interviews. Individual interviews were conducted by SR in a private location at ANGAU hospital and recorded using Zoom, version 6.1 (Zoom Video Communications, San Jose, California, USA). SR made notes during and after the interviews. Data were predominantly transcribed by SR with limited use of Zoom automatic transcription software (due to observed inaccuracies). Given the use of snowball sampling, it is not possible to determine the number of people invited to participate in the study. A single participant chose to withdraw before the interview began. No repeat interviews were conducted. Given the hospital’s limited printing and electronic infrastructure, it was not possible to securely return transcripts to participants.

### Data analysis

We used reflexive thematic analysis to analyse transcripts in accordance with the steps outlined by Braun and Clarke [20]. SR inductively coded all transcripts using NVivo, version 15 (Lumivero, Denver, Colorado, USA), creating candidate themes. These themes were discussed among the research team to develop subthemes. Specifically, researchers FF, WA, TA, NB, and DY offered invaluable local contextual perspectives.

### Context

This study was part of a quality-improvement collaboration at ANGAU hospital in Lae, PNG. The collaboration was between the ANGAU hospital’s paediatrics department and Monash Children’s Simulation.

The predominant staffing groups in the paediatrics department are nursing staff, community health workers (CHWs), health extension officers, and doctors. This is despite the typical association of CHWs and health extension officers with providing care in the community and rural areas [21,22]. Each staffing group is typically responsible for completing patient documentation relevant to the clinical care they provide. For example, doctors are expected to record patient progress notes, while nurses are expected to document vital signs and nursing notes. Clinical documentation is paper-based with no substantial integration of electronic health records. When conducting data collection for audit or research purposes, the group responsible will depend on the nature and organisation of the project.

The quality improvement initiative relevant to this study involved introducing simulation-based education and online learning for paediatric healthcare workers. One focus of the programme was acute paediatric care, specifically malnutrition, and dehydration due to gastroenteritis. This included planning a clinical audit for evaluation. Malnutrition and dehydration are significant challenges in PNG. The World Bank estimated in 2010 that 49.5% of children under five suffered from stunting [23], while diarrhoeal disease is a leading cause of death in children in this setting [24].

The audit was designed as a prospective data collection over six weeks, pre- and post-programme. Preliminary data would be collected for all paediatric patients presenting to the hospital, and then detailed data would be collected for patients with dehydration and malnutrition. Data collection was initially led by nursing staff with additional participation of other healthcare workers as available. Data collected included basic patient demographics, management and outcomes. However, several challenges required ANGAU hospital faculty to adjust their data collection approach. Rather than collecting data at the time of treatment, data were instead collected from patient records at the conclusion of the day or week. The clinical audit findings are beyond the scope of this study.

### Research team and reflexivity

Reflexivity, which involves acknowledging subjectivity as part of the research process, is essential in qualitative research [25]. This research was conducted by a diverse team of healthcare workers and researchers from Australia and PNG who have a history of collaboration. We have incorporated this plurality of perspectives into our analysis. SR has conducted research in the Pacific Island context for several years, including coordinating data collection at ANGAU hospital. These experiences likely influenced the lens through which he conducted this research. Being from Australia likely created an unequal power dynamic between SR and the participants. SR attempted to minimise the impact of this by creating a relaxed environment and clarifying the shared goals of improving paediatric care at ANGAU hospital. A complete reflexivity statement addressing all authors is available in Appendix S2 in the **Online Supplementary Document**.

## RESULTS

### Participant demographics

We interviewed 20 participants, of whom the majority were nurses (**Table 1**). Interviews typically lasted between 20–40 minutes.

**Table 1:** Characteristics of interview participants

Demographic	n (%)
Gender	
*Woman*	15 (75)
*Man*	5 (25)
Occupation	
*Nurse*	15 (75)
*Health extension officer*	2 (10)
*Doctor*	1 (5)
*Nurse-midwife*	1 (5)
*Community health worker*	1 (5)
Years worked in the profession	
*0–5*	7 (35)
*5–10*	2 (10)
*10–15*	2 (10)
*15–20*	3 (15)
*>20*	6 (30)
Previous participation in any research/audit	
*Yes*	16 (80)
*No*	3 (15)
*Unsure*	1 (5)
Previous participation in a recent paediatric audit	
*Yes*	14 (70)
*No*	6 (30)

### Overview of themes

We conceptualised five themes and eight subthemes from the data (**Table 2**), while illustrative quotes are presented in-text (Appendix S3 in the **Online Supplementary Document**).

**Table 2:** Themes and subthemes conceptualised in a study of factors affecting paediatric clinical audit data collection in Papua New Guinea

Themes	Subthemes
Creation of a quality improvement culture	Staff motivation to improve data collection; Broad engagement of staff; Creation of a safe environment for patients to share data; The importance of institutional support
Staff workload	
Current practice of documentation	
Research infrastructure	Staff training and supervision; Physical resources and space
Study procedures	Staff preparation; Collection tool design

### Theme: creation of a quality improvement culture

#### Staff motivation to improve data collection

Most participants were highly motivated to improve data collection processes in their workplace. Benefits for clinical practice and patient care were often highlighted, with recognition of potential improvements at the systems level (*e.g.* audits) and in individual clinical practice. Staff were particularly interested in data collection to highlight practices and outcomes needing improvement. Others described how data could be used to advocate for change and secure funding from organisations such as the United Nations:

We need information to see what is wrong. So we can make decisions. *–* *Participant 12.*

This data will help improve my practice, so I have to collect the right information. *– Participant 14.*

Everyone has been talking about evidence-based medicine… where’s my data?... It also tells the funders out there… like UNICEF. *– Participant 19.*

#### Broad engagement of staff

Participants emphasised the importance of involving staff across all seniority levels and disciplines in data collection processes. It was viewed as essential to highlight the purpose of the audit or research to these individuals. Staff were subsequently more motivated to engage and participate. Engaging senior staff in clinical leadership roles was seen as crucial for organising projects and fostering interest among junior staff. Direct involvement of junior staff, particularly those responsible for data collection, was also beneficial. Additionally, interdisciplinary collaboration played a key role in fostering an effective and constructive research environment:

That’s a common understanding…[that] we’re doing it for someone’s betterment… I never realised that it’s good for the Department. *– Participant 1.*

When we see our senior staff doing some research, then it will… send some inspiration.* – Participant 12.*

If they knew that these data were going to be used for bringing funds, or bringing more studies, [or] to do [make] changes… they will ensure that the data is collected very well. *– Participant 20.*

#### Creation of a safe environment for patients to share data

Concerns were raised about the quality of patient-provided data, particularly sensitive information such as disease status. Participants reported that patients would avoid disclosing prior hospital presentations or diagnoses, thereby complicating management. This was attributed to a lack of trust in patient confidentiality. Staff recognised a need to adjust their practices to collect sensitive data effectively and confidentially:

Some of the questions were not answered because of the patients not cooperating with us.* – Participant 7.*

We have to get into the consultation room or where there is a private [room]. And then collect all this…* – Participant 15.*

Sometimes they try to hide the previous history… They don’t want us to see their previous history… the HIV patients. *– Participant 17.*

#### The importance of institutional support

Some senior staff or management had historically expressed concern regarding data collection. In particular, participants spoke about individuals who worried that data collection would reflect poorly upon them or their institution. Another reported that their research ethics committee had been inactive, which impeded their initiation of research:

It can paint good pictures… or it can also paint bad pictures about the hospital*.* *– Participant 19.*

We need the hospital to have a function[ing], effective research committee… This will encourage us to… collect data and conduct research in the hospital. *– Participant 12.*

### Theme: staff workload

The majority of participants discussed staff workload as an issue, stating that they lacked sufficient staff to manage their clinical case load. This meant they struggled to collect clinical data, particularly at the time of patient presentation. Participants also referenced a noticeable increase in patients during the paediatric audit period. In many cases, workforce challenges meant individuals had to choose between patient care or data collection. Conversely, several staff viewed data collection as very achievable if they had access to greater staffing numbers. This theme was particularly significant, as it seemed to impact on other areas such as regular clinical data documentation:

*It’s the big ward... and if doing such work it’s really tired [tiring]. Yeah. Because 45 patient[s]… [are being cared for] by myself and CHW [Community Health Worker].* *– Participant 6.*

Who’s going to take care of the patient… while I’m doing the collecting of data? *– Participant 15.*

If… there’s a lot of patient[s] there… how would you attend to the patients? And then, you will [be] wasting, taking time filling in the form while the other patients are complaining, or one is going into arrest. *– Participant 3.*

### Theme: current practice of documentation

There was suggestion that standard practices of documentation impeded collection of high-quality data. Several participants described challenges with missing data in medical records when attempting data collection. Participants were frustrated and expressed that it should have been documented at the time of patient care, both for the benefit of the patient and to ensure accurate research data. Others discussed how they believed essential procedures were not being conducted and therefore documented, such as vital observations. They attributed this predominantly to a lack of time, but also to a lack of awareness and standards. Several participants spoke about the data collection form itself being incorporated into clinical practice and used to guide management in lieu of regular documentation:

How can we collect data when we don’t see the actual thing in the charts. So, that’s… my struggle that I faced while collecting data. *– Participant 2.*

The vital observations are very important. The measurements are not being done. And it, the history, is not properly being taken. *– Participant 17.*

During the ward round and all this, the doctors also used this data collection form to see the initial treatment that was given. *– Participant 8.*

### Theme: research infrastructure

#### Staff training and supervision

Participants expressed that opportunities to receive training in research and data collection would be valuable for improving interest and accuracy. Additionally, several participants wanted more supervision and support for their own research projects. The nature of the desired support varied between individuals. Junior participants with no audit or research experience tended to express general interest in training across all aspects of these processes. Alternatively, senior staff requested more targeted training, such as in statistical analysis and presentation:

If only we have like, more training… involving everyone… That would help all of us.* – Participant 2.*

Research has to be part of our curriculum. *– Participant 1.*

If I were to do something major, I would prefer a proper, someone who really knows how to do this to come in and help us, especially with data analysis. *– Participant 4.*

#### Physical resources and space

Participants encountered multiple resourcing challenges while collecting clinical data. Printing and photocopying for paper-based collection were often difficult due to ink and paper shortages, while digital collection was hindered by inadequate computer systems. There was a strong desire for a dedicated research space to enable secure, efficient data collection. One participant expressed a desire for computer-based data collection to maximise efficiency and data accessibility:

We had issues with the [printer] inks… we ran out of the forms. *–* *Participant 17.*

If we are to collect data, it has to be hard copy. Not a lot of us have laptops, computers… I know every ward had one computer each, [but] it has a lot of viruses or it has crashed. *– Participant 4.*

Every ward in the hospital should have allocated… a[n] office, where every [all] staff come in for their shifts, they have to collect [data]. *– Participant 15.*

### Theme: study procedures

#### Staff preparation

For any study, participants highlighted the need for adequate preparation. It was noted that staff often rotated, meaning even if an initial briefing had been provided, new staff could be unaware of the data collection process. Participants discussed the value of establishing a designated role or team responsible for ongoing data collection:

We had staff rotated, you know. So, some know what to do, some don’t. They are not familiar. They have not been told. *– Participant 17.*

If they [new staff] are being made aware… then I think everybody would be able to [collect data]. *– Participant 17.*

There should be another team set up for this. So this will be effective. When we want to collect the data, you just go to that [those] specific people. *– Participant 15.*

#### Collection design

Many comments were provided by participants regarding the data collection tools. Participants spoke positively about having a targeted form that was easy to use. The choice of which data to collect was also relevant. Broadly speaking, participants were satisfied with the paediatric audit given the significant challenges posed by dehydration and malnutrition. However, there remained areas for improvement, highlighted by a suggestion to include measurement of the mid-upper arm circumference (MUAC):

[The form is] not complicated... it’s clear. [You] can just tick, tick, and circle. *– Participant 2.*

That was a nice topic… because we have a lot of SAM [severe acute malnutrition] patients here.* – Participant 16.*

Here, the MUAC here is very important… it’s not in the form. *– Participant 2.*

## DISCUSSION

In this research, we outline numerous factors which affect the collection of clinical data at a tertiary centre in PNG. The findings will inform future data collection at ANGAU hospital and will be relevant to comparable low-resource settings.

This study highlights the role of a quality-improvement culture in successful data collection. Despite considerable workforce pressures, high levels of staff motivation contributed to the successful paediatric audit. Engaging staff across all seniorities and disciplines was seen as essential for effective data collection. This was a valuable insight, given the emphasis in the existing literature on local champions [3]. The ‘local champion’ model has been discussed in the context of global health research, and involves identifying and recruiting individuals to drive engagement [26]. However, while our local leaders were extremely important to this study, participants suggested that relying solely on senior staff is insufficient. In addition to junior staff involvement, participants expressed a desire for greater institutional support for data collection. Overall, this is consistent with the notion of distributed leadership, where responsibility for task completion, in this case, data collection, is shared [27]. It also aligns with USAID’s assertion that a data-use culture should ‘promote participation and inclusiveness between stakeholders’ [14].

Reports of patient non-disclosure of sensitive information are concerning, given the relevance of co-morbidities to optimal patient care [28]. At ANGAU hospital, clinicians are expected to uphold patient confidentiality; however, the hospital’s significant caseload poses a challenge to maintaining privacy. This is a known challenge in crowded healthcare settings [29]. The reluctance of people living with HIV to share their previous history also points to the ongoing stigma of the diagnosis in PNG [30]. While the causes of patient confidentiality concerns are likely multifactorial, they indicate a need for targeted education and clear regulations for healthcare workers. This may improve the therapeutic relationship and ensure patients with known co-morbidities are treated appropriately.

Staff workload was reported to significantly impede data collection. Contextually, this is unsurprising given the known workforce challenges in this setting [31]. However, this study provides insights into how these workforce shortages impact staff, who must choose between effective patient care and data collection. Moreover, participants’ experiences with the paediatric audit demonstrated how data collection is vulnerable to sudden changes in workload. This is indicative of a limited surge capacity, which describes the ability of a healthcare system ‘to respond to a sudden increase in patient care demands’ [32]. In the context of clinical data collection, limited surge capacity may indicate a need for flexibility in data collection processes or to establish contingency plans for unexpected events. For our own paediatric audit, we collected data from patient records rather than during clinical care. Another potential approach to circumvent clinical surges, if acceptable to the hospital and feasible, would be to employ paid data collectors who do not provide clinical care.

While shifting to data collection from patient records was necessary for our audit, this experience also revealed additional challenges for staff. Standard documentation practices did not always include critical values, such as initial clinical observations. This highlights how patient care practices are closely interrelated with audit and research capacity. Without effective systems for documenting patient care, audit and research processes using real-world data are significantly hindered [33]. In the ANGAU hospital context, the lack of documentation practices also raises concerns regarding how patients are monitored throughout their admission, and may suggest that further training is required. Despite these challenges, the relationship between clinical documentation and data collection initiatives can also be used to the advantage of clinicians and researchers. The data collection in our paediatric audit was incorporated directly into clinical practice to improve patient care. Future audits and research projects may be designed with these dual purposes in mind. For example, it may be possible to design a training programme to improve both clinical documentation practices and the incorporation of data into regular clinical audits.

This study also highlights the need for an improved research ecosystem to enable effective data collection. There was demand for external expertise in training and supervision, suggesting the possibility of further collaborations. This is a known benefit of global health collaborations; however, such initiatives should be mindful of research equity and data sovereignty [34,35]. Tangible resources taken for granted elsewhere, such as printing, were not always available at the hospital. Future initiatives should strongly consider providing the necessary resources to overcome these barriers. Given the global shift towards electronic health records, these are a promising method of overcoming paper-based resource challenges and improving real-world data collection and use. However, given the current state of digital literacy and the electricity grid in PNG [36,37], we would be cautious in recommending this transition without significant planning for introducing and sustaining the service.

It was evident from the interviews that the data collection procedures of any research or audit project had significant impacts. This theme was highly informed by participants’ experiences with our own paediatric audit. It was clear that many participants felt they lacked sufficient information about the study, which should be addressed in future studies. On the other hand, the design of the form was viewed favourably, which we attribute to the collaborative involvement of local clinicians and Australian clinical researchers. The design of any future data collection is likely to be project-dependent. In a context such as this, data collection tools should be designed to impose minimal impact on staff workload. For our study, this involved selecting basic variables such as vital signs, initial treatment details and simple outcome measures (*e.g.* length of stay, mortality). However, opportunities for improvement were still highlighted. It seems likely that using MUAC measurements would have been more effective than relying on birth data and percentiles to assess malnutrition.

The involvement of healthcare workers from different areas within the department under study, with varying degrees of experience, is a strength of this study. For the sampling, we were guided by information power rather than data saturation, in accordance with established practice [38]. We felt this sample was sufficient to address our research question, particularly given the quality of the dialogue.

A limitation of this study is that the paediatric audit was often participants’ first experience collecting clinical data for either audit or research, and a small number had never completed any data collection. This led to some difficulty asking questions about potential future data collection initiatives. However, we ultimately believe the audit aided less-experienced participants to understand what such an initiative could look like. Another limitation was the use of snowball sampling, which has the potential to skew the interviewed participants. However, there was no obvious alternative sampling method feasible in this setting, and we felt our sample was sufficient to capture a variety of perspectives. The sample was predominantly composed of nurses, and it is possible that other professions face similar or different challenges when conducting data collection. Finally, the interviews were conducted in English. While all participants were fluent in English, there were occasional instances where participants wanted to speak Tok Pisin. cultural nuances may therefore not have been fully explored in certain interviews. This may be a focus of future studies, as may be investigations of how staff in different disciplines and positions of seniority perceive and experience data collection.

## CONCLUSIONS

Ultimately, this study offers valuable insights for future data collection initiatives in similar settings. Staff at ANGAU hospital were highly interested in improving clinical data collection but faced several barriers to doing so. Strategies should be implemented to address challenges related to staff workload and resource limitations, such as compensating data collectors and ensuring access to necessary research equipment. Training in documentation before data collection initiatives should be prioritised and could provide additional benefits to clinical care. Similarly, comprehensive staff education should be conducted before launching projects, ideally accompanied by a pilot data collection phase. Strong staff engagement and the potential benefits of data collection suggest that further efforts to refine and support these processes are warranted. Given the critical role of data collection in public and global health, these findings have broader implications for strengthening health systems at both regional and global levels.

## Additional Material


Online Supplementary Document
