# Supplementary material for: Factors affecting the collection of clinical data for quality improvement at a tertiary centre in Papua New Guinea: a qualitative study
Source: J Glob Health. 2026 May 8;16:04125. doi: 10.7189/jogh.16.04125 (PMC13154336; doi:10.7189/jogh.16.04125)
Supplement: Online Supplementary Document [file jogh-16-04125-s001.pdf]

Supplement to: : Robinson SJA, Failing F, Albert W, Buni N, Yema D, Anterea T, Nestel D, McLeod E, Pacilli M, Nataraja RM. Factors affecting the collection of clinical data for quality improvement at a tertiary centre in Papua New Guinea: a qualitative study. J Glob Health. 2026;16:04125.

Appendix S1. COREQ (COnsolidated criteria for REporting Qualitative research) Checklist

A checklist of items that should be included in reports of qualitative research. You must report the page number in your manuscript where you consider each of the items listed in this checklist. If you have not included this information, either revise your manuscript accordingly before submitting or note N/A.

Topic	Item No.	Guide Questions/Description	Reported on Page No.
**Domain 1: Research team and reflexivity**			
Personal characteristics			
Interviewer/facilitator	1	Which author/s conducted the interview or focus group?	
Credentials	2	What were the researcher's credentials? E.g. PhD, MD	
Occupation	3	What was their occupation at the time of the study?	
Gender	4	Was the researcher male or female?	
Experience and training	5	What experience or training did the researcher have?	
Relationship with participants			
Relationship established	6	Was a relationship established prior to study commencement?	
Participant knowledge of the interviewer	7	What did the participants know about the researcher? e.g. personal goals, reasons for doing the research	
Interviewer characteristics	8	What characteristics were reported about the inter viewer/facilitator? e.g. Bias, assumptions, reasons and interests in the research topic	
**Domain 2: Study design**			
Theoretical framework			
Methodological orientation and Theory	9	What methodological orientation was stated to underpin the study? e.g. grounded theory, discourse analysis, ethnography, phenomenology, content analysis	
Participant selection			
Sampling	10	How were participants selected? e.g. purposive, convenience, consecutive, snowball	
Method of approach	11	How were participants approached? e.g. face-to-face, telephone, mail, email	
Sample size	12	How many participants were in the study?	
Non-participation	13	How many people refused to participate or dropped out? Reasons?	
Setting			
Setting of data collection	14	Where was the data collected? e.g. home, clinic, workplace	
Presence of non-participants	15	Was anyone else present besides the participants and researchers?	
Description of sample	16	What are the important characteristics of the sample? e.g. demographic data, date	
Data collection			
Interview guide	17	Were questions, prompts, guides provided by the authors? Was it pilot tested?	
Repeat interviews	18	Were repeat inter views carried out? If yes, how many?	
Audio/visual recording	19	Did the research use audio or visual recording to collect the data?	
Field notes	20	Were field notes made during and/or after the inter view or focus group?	
Duration	21	What was the duration of the inter views or focus group?	
Data saturation	22	Was data saturation discussed?	
Transcripts returned	23	Were transcripts returned to participants for comment and/or	

Topic	Item No.	Guide Questions/Description	Reported on Page No.
		correction?	
**Domain 3: analysis and findings**			
Data analysis			
Number of data coders	24	How many data coders coded the data?	
Description of the coding tree	25	Did authors provide a description of the coding tree?	
Derivation of themes	26	Were themes identified in advance or derived from the data?	
Software	27	What software, if applicable, was used to manage the data?	
Participant checking	28	Did participants provide feedback on the findings?	
Reporting			
Quotations presented	29	Were participant quotations presented to illustrate the themes/findings? Was each quotation identified? e.g. participant number	
Data and findings consistent	30	Was there consistency between the data presented and the findings?	
Clarity of major themes	31	Were major themes clearly presented in the findings?	
Clarity of minor themes	32	Is there a description of diverse cases or discussion of minor themes?	

Developed from: Tong A, Sainsbury P, Craig J. Consolidated criteria for reporting qualitative research (COREQ): a 32-item checklist for interviews and focus groups. *International Journal for Quality in Health Care*. 2007. Volume 19, Number 6: pp. 349 – 357

Once you have completed this checklist, please save a copy and upload it as part of your submission. DO NOT include this checklist as part of the main manuscript document. It must be uploaded as a separate file.

Appendix S2: Reflexivity Statement and questions

Reflexivity Statement

This research was conducted by a diverse team of healthcare workers and researchers from Australia and PNG whose institutions and members have a long history of collaborating. We have incorporated this plurality of perspectives into our analysis and interpretation.

At the time of this research, SR was a male PhD candidate at Monash University, Australia. He had conducted research in the Pacific Island context for several years, which included coordinating data collection at ANGAU Hospital, Lae. Through this process, he worked previously with several of the interviewees. These experiences likely influenced the lens through which he conducted this research. Being from Australia, a high-income country, likely created an unequal power dynamic between SR and the research participants. SR attempted to minimise the impact of this on the interview by creating a relaxed environment for participants and clarifying the shared goals of improving paediatric care at ANGAU Hospital. Being a medical student also reduced the power differential between SR and research participants.

FF, WA, TA, NB and DY are senior healthcare workers at ANGAU Hospital. In addition to being part of the research team for this qualitative study, they participated as interviewees. However, their research involvement occurred after their interviews, meaning it is unlikely to have influenced their responses. Separately, the prior experiences of FF, WA, TA, NB and DY collecting clinical data for the paediatric audit is likely to have shaped their contributions to the research.

Finally, DN, MP, EM and RN are researchers, with expertise variously in global health, education and as healthcare professionals in Australia. Their involvement is likely to have been similarly influenced by power dynamics relating to the collaboration between a high-income country and a lower-middle-income country.

Appendix S3: Themes and subthemes conceptualised from the data with comprehensive indicative quotes

Themes	Subthemes	Quotes:
**Creation of a culture of quality improvement**	**Staff motivation to improve data collection**	"It's important because… it would be much more helpful for the hospital, and also the government, to address some of these issues too, so like it's very important collecting these datas." (Interviewee-3)<br><br>"I came across the benefit of data collecting… like in paediatrics we are facing huge shortage of TB drugs… NGO provide[s] us with TB treatment or TB drugs… they gave us drug[s] according to the statistics… When there's not enough statistic collected, [there's] not enough supply for the drug." (Interviewee-10)<br><br>"We need data to convert data into information. And yes, we need information to see what is wrong. So we can make decisions based on the information…" (Interviewee-12)<br><br>"We should feel responsible to at least contribute. Like, we have to be willing to help, and allow this kind of research to take place" (Interviewee-12)<br><br>"It is important. It's very important… from that we can, we can see how well we performed to the, to our patient. And that way, too, we can lessen the mortality rate." (Interviewee-13)<br><br>"I want to do more cause, it really boost[s] us to continue to do this. That's why I'm happy to come and interview, you to interview. Cause, I want to do more of this data collection." (Interviewee-13)<br><br>"My view is that, this data will help improve my practice so I have to collect the right information." (Interviewee-14)<br><br>"The number of SAM patients, each month that coming in…it will put some light in this… we'll see the result that ANGAU really needs a malnutrition ward of its own." (Interviewee-16)

Themes	Subthemes	Quotes:
		"It helps us identify the number of cases that we are seeing… and maybe help us, how we would improve" (Interviewee-17)<br><br>"Everyone has been talking about evidence-based medicine… where's my data? Where's my proof? So like data collection is very important. It also tells the funders out there… those agents who come in to support us here." (Interviewee-19)
	**Broad engagement of staff**	"That's a common understanding I think that everyone knows about research like it, we're doing it for someone's betterment for someone else. I never realised that it's good for the Department." (Interviewee-1)<br><br>"We would like [to] have more research upon this, but because there is not enough good data collection. So we are just, honestly speaking, we are just coming to work and going home. Just the routine work only." (Interviewee-2)<br><br>"We nurses don't really participate in collecting information on a lot of things" (Interviewee-4)<br><br>"Our doctors sat down, saw that [presentation of data]. And everyone agreed after that, we will all help." (Interviewee-4)<br><br>So [our Nurse Unit Manager] have to remind us [about data collection] every time, everyday." (interviewee-10)<br><br>"In our setting… everyone is not equipped with how to collect data" (Interviewee-11)<br><br>"When we see our senior staff doing some research, then it will, you know, send some inspiration. Motivate us to, you know, do our research proposal." (Interviewee-12)<br><br>"I felt that, like my presence, being there all that time also would help in making sure that the forms were filled correctly." (Interviewee-17)

Themes	Subthemes	Quotes:
		"Those who have, done studies that, involve data collection, were in better position to understand what we were saying." (Interviewee-20)<br><br>"If they knew that these data were going to be used for bringing funds, or bringing more studies, [or] to do changes and all this one, they will ensure that the data is collected very well." (Interviewee-20)
	**Creation of a safe environment for patients to share data**	"They sometimes will give false information…" (Interviewee-6)<br><br>"Sometimes they will still give us a false information… It's really, difficult on that side when collecting data so." (Interviewee-6)<br><br>"Some of the questions were not answered because of the patients not cooperating with us" (Interviewee-7)<br><br>"Either the patient or the staff, that you want them to help, you have to get their, consent first." (Interviewee-9)<br><br>"We're always, go for the consent and like, it's confidential for our patient… But then if there's, others which, [are] apart from the medical background... While collecting data there's some sensitive cases coming in. So it's more risk… [if they] knows the patient, they will easily, like, pass out information." (Interviewee-11)<br><br>"Some patients they just, you know, they are on their way home… if you want to ask questions or if you want to collect data then, they will say, 'We don't live here'." (Interviewee-12)<br><br>"In the cases where there is a, the condition of the patient, maybe it's too personal… sometimes patient will not give you the fact information, or true." (Interviewee-15)

Themes	Subthemes	Quotes:
		"You have to assure the patient and then, have empathise, or empathy. That you have to put yourself into the patient's situation and then, you have to talk to them. So in this way they're going to give you every [all the] information that you needed. So, these are some of the things that we improve. On data collection." (Interviewee-15)<br><br>"We don't have to put the patient in a general ward and then collect data. To collect all this, see the data from the patients, we have to get into the consultation room or where there is a private [room]. And then collect all this data so patient will have a, open up and talk to you, and give every information so." (Interviewee-15)<br><br>"A lot of times, they attend the clinics, that sometimes they try to hide the previous history… They don't want us to see their previous history… the HIV patients" (Interviewee-17)
	**The importance of institutional support**	"We need the hospital to have a function[ing], effective research committee to be in place. This will encourage us to, you know, collect data and conduct research in the hospital." (Interviewee-12)<br><br>"So like they consider what is very important first. Then data collection is like, they don't really consider it like it's important." (Interviewee-18)<br><br>"The recommendation part of it. Yeah. There are some recommendations that is achievable. And there are some recommendations that, that is like, it's not achievable… Like, in terms of, like, resource… one of our nurses did a presentation. And she recommended [something]… but they cannot give us." (Interviewee-18)<br><br>"When there is a good data you get good response… From the management… Sometimes you get good response, sometimes nothing [when there's bad data]." (Interviewee-18)<br><br>"I gave up because… maybe my data is not really good. That is all, people up there might not want my recommendation." (Interviewee-18)

Themes	Subthemes	Quotes:
		“As long as we explain it to our staff and our management. The management knows that we are doing this for the good of our, of our patients.” (Interviewee-19)<br><br>“Things may become published later on… it can paint good pictures out there. Yeah, nationally or internationally. Or it can also paint bad pictures about the hospital… Any hospitals in the country. They're so sensitive about what is being published out there.” (Interviewee-19)<br><br>“Just the publications that make the hospital and the management think ‘Oh, so how have they been managing?’” (Interviewee-19)
**Staff workload**		“If… there’s a lot of patient[s] there. Then it is difficult for you to. How would you attend to the patients? And then, you will [be] wasting, taking time filling in the form while the other patients are complaining, or one is going into arrest.” (Interviewee-3)<br><br>“The total number of patients that are coming in one day… it doesn’t decrease. It doesn’t decrease… the number increases… every morning when I come to work, I see that same number. There’s no changes in [it]… Like, we are trying to decrease the number of people coming in, while its number is still increasing. So. I would say that it’s quite challenging too.” (Interviewee-3)<br><br>“It’s the big ward, and if me, by myself, with the CHW, and if doing such work, it’s really tired. Yeah. Because 45 patient and, if [they are being cared for] by myself and CHW. CHW will carry their own work according to his or her JD, job description.” (Interviewee-6)<br><br>“You nursing officers, you won’t sit. You’re gonna stand til daybreak if you’re working night… so it’s a busy ward at Paediatric.” (Interviewee-6)<br><br>“The patients and the guardian… if you are too slow, they will shout at you. So, like, with that in mind, sometimes we forgot to fill the data collection form.” (Interviewee-8)

Themes	Subthemes	Quotes:
		"With data collection, it's time-consuming. And then, when we delay, the staff [are] home very late and they complain. So the nurses they don't come early. They say 'We went home late, so'." (Interviewee-12)<br><br>"So like, I think that it's, like, times when less number of patients come, it, it will be at least okay to, you know, sit down and fill in the data." (Interviewee-12)<br><br>"If I am only one working in the afternoon shift, then who's going to take care of the patient and then while I'm doing the collecting of data?" (Interviewee-15)<br><br>"I am taking care of twenty [patients], and I'm the only one working. So ANGAU should recruit more staffs. So if there's enough manpower, those extra work after hours we can just, help whoever want to do the data collection. We just help them to collect the data" (Interviewee-15)<br><br>"We had a[n] increased number of patients compared to the previous years. At that time there was so many patients, and especially the admitted patients. There were so many admitted patients. And so the bed number in the ward, the beds in the ward could not cater for all the admissions. And so we had to have patients admitted in the outpatient. And, patients, even Outpatient too, could not cater for all the admissions. So we had patients, two to three patients being put on one bed." (Interviewee-17)
**Current practice of documentation**		"How can we collect data when we don't see the actual thing in the charts. So, that's one of the… like my struggle that I faced while collecting data." (Interviewee-2)<br><br>"Collecting the information, it's kind of hard to [do], because the information is all over the place." (Interviewee-3)<br><br>"If only that data were labelled out in a, I mean proper way at the time of admission. Then that would be easy for us to collect that data." (Interviewee-3)<br><br>"It help[s] us to, diagnose the patient, yeah… assessing of the patient, quick." (Interviewee-7)

Themes	Subthemes	Quotes:
		"If he or she used the data collection, then during the ward round and all this, the doctors also used this data collection form to see the initial treatment that was given." (Interviewee-8)<br><br>"After I did collect the data from this, using the, this malnutrition sheet. I tend to realise, there's some things that are missing… we should have collect those kind of data" (Interviewee-11)<br><br>"If we do what we're supposed to do, it will be easy for us to collect that data." (Interviewee-13)<br><br>"[The] past admission officer that admitted that patient have [has] to collect full data from that patients… So. At the end, we can collect that data for research and all that. And it will help us." (Interviewee-15)<br><br>"Sometimes they do, sometimes. But then with evidence, it should show that we have done. If we're not recording then that means that, like we are not doing anything." (Interviewee-16)<br><br>"I'm also picking up some things which, that they are not doing… the vital observations are very important. The measurements are not being done. And it, the history is not properly being, taken." (Interviewee-17)
**Research infrastructure**	**Staff training and supervision**	"We have to get people to really understand importance of research and audit and this, it's for the good of our practice and our Department." (Interviewee-1)<br><br>"Research has to be part of our curriculum." (Interviewee-1)<br><br>"If only we have like, more training, involving a lot, involving everyone... That would help all of us. To feel the confidence… Like all of us have this potential." (Interviewee-2)<br><br>"Please, can you and the team talk more about the statistics? So we can like, collect the statistics like, good, accurate statistics. And yeah, we won't miss out on any data." (Interviewee-2)

Themes	Subthemes	Quotes:
		"If we had specific workshops on audit itself, research itself… for those of us who will be participating in this… it'll be nice to give them refresher courses or something. Just a workshop, just a refresher and…" (Interviewee-4)<br><br>"With research it would definitely be someone to supervise the research. Who would, you know say, yes, you're doing this correctly, you're doing this incorrectly. So proper supervisor. We can do it, we just need supervisors. And with audit the same." (Interviewee-4)<br><br>"If I were to do something major, I would prefer a proper, someone who really knows how to do this to come in and help us, especially with data analysis. And the presentation itself." (Interviewee-4)<br><br>"If you tend to leave us, we will leave everything as is. So "How far are you?" and things like that. And, little reminders, and, that, that will help." (Interviewee-4)<br><br>"I filled out one of those request forms and specifically asked if we can have a nurse educator who will just concentrate on research." (Interviewee-4)<br><br>"I haven't started an audit myself yet… So, somebody assisting or somebody trying to, we can work together." (Interviewee-9)
	**Physical resources and space**	"We had printing issues so we didn't print a hard copy of the approval letter [for another study]" (Interviewee-4)<br><br>"If we are to collect data, it has to be hard copy. Not a lot of us have laptops, computers… I know every ward had one computer each, [but] it has a lot of viruses or it has crashed or something" (Interviewee-4)<br><br>"If we can allocate a little section where, a cupboard or something… so you know your documents are safe and nobody's tampering with them" (Interviewee-4)

**Themes**	**Subthemes**	Quotes:
		"Every ward in the hospital should have allocated… a[n] office. Where every [all] staff come in for their shifts, they have to collect [data]" (Interviewee-15)<br><br>"I prefer [if we had a] computer because, it will minimise time consuming. And it will also, accessible to the data." (Interviewee-15)<br><br>"In printing, we had issues with the inks as well. So, sometimes when we ran out of the forms…" (Interviewee-17)<br><br>"We have an issue with our binders… they are sometimes inconsistent with supplies. So, we don't have the binders." (Interviewee-17)<br><br>"The supply is not consistent so… that's another difficulty, yeah. With the inks." (Interviewee-17)<br><br>"The clerks don't have a place to sit, so they just, just like I don't have an office, the clerks too just sit anywhere" (Interviewee-17)<br><br>"I need[ed] to have bigger size paper to, just to capture all the [birth] weights." (Interviewee-18)
**Study procedures**	**Staff preparation**	"Get a time for everyone to meet, briefing for everyone, so that we understand why we have to fill [the forms]." (Interviewee-1)<br><br>"If you really put someone in charge of this, okay this your dedicated responsibility, you do the upkeep for this book… you'll get a better, better work that way than living it open for everyone who's working on duty to come in." (Interviewee-4)<br><br>"There are some staff who are really interested in little things. If you say, okay you're part of the audit team, to them that is a huge boost" (Interviewee-4)<br><br>"Yeah, there should be some people coming and telling us about the form, before we actually filling it. We should be informed and, how to do it, what to fill." (Interviewee-7)

Themes	Subthemes	Quotes:
		"It will be more helpful if everyone is taught how to collect data. Then it's easier for them." (Interviewee-11)<br><br>"There should be another team set up for this. So this will be effective. When we want to collect the data, you just go to that [those] specific people." (Interviewee-15)<br><br>"They should allocate the officer or someone who has to do and record. Somebody to be in charge of doing that and others to assist or seeing them doing it." (Interviewee-16)<br><br>"Have the researcher on the ground. Yeah. That, something that I see would help in, with the data collection. So like, every time you are coming and you are checking." (Interviewee-17)<br><br>"Every day there's a new staff coming on. So, if they are being made aware of, that we have a survey going on, then I think everybody would be able to." (Interviewee-17)<br><br>"We had staff rotated, you know. So, some know what to do, some don't. They are not familiar. They have not been told." (Interviewee-17)<br><br>"Having like, not one session, but at least two sessions or three sessions telling them about the importance of the study. And they must understand what it is for." (Interviewee-19)
	**Collection design**	"[The form is] not complicated… it's clear. [You] can just tick, tick, and circle." (Interviewee-2)<br><br>"The form was alright but that part, the birthing part… I wasn't able to fill in that part." (Interviewee-5)<br><br>"Here, the MUAC here is very important… it's not in the form." (Interviewee-2)<br><br>"It's perfect data form yes. I would say it's perfect… The information that we're supposed to fill in… they were all in the form." (Interviewee-3)

Themes	Subthemes	Quotes:
		"You get someone that's like on the ground to read that, because English is not our language… if need be, change one or two words, the meaning is the same. Just change one or two words so locally, we understand it." (Interviewee-4)<br><br>"The questions are already written in the paper, so it makes it easy to ask them what was written." (Interviewee-8)<br><br>"I think you've picked… the very cases that we deal with. And that's a very good topic." (Interviewee-12)<br><br>"The data collection form is formatted in a way that we can easily read and understand." (Interviewee-14)<br><br>"To effectively collect data in a short period of time… you have to design a form that will not consume a lot of time… So, you just have to stay down on the general and necessary things, that should feed into the research." (Interviewee-15)<br><br>"That was a nice topic that you have taken on for your studies… because we have a lot of SAM patients here." (Interviewee-16)<br><br>"It had to capture those things that we needed… we cannot leave out those [that] information." (Interviewee-19)
